# Does Excellence Correspond to Universal Inequality Level?

**DOI:** 10.3390/e27050495

**Published:** 2025-05-02

**Authors:** Soumyajyoti Biswas, Bikas K. Chakrabarti, Asim Ghosh, Sourav Ghosh, Máté Józsa, Zoltán Néda

**Affiliations:** 1 Department of Physics, SRM University-AP, Amaravati 522240, India; sourav_ghosh@srmap.edu.in; 2 Department of Computer Science and Engineering, SRM University-AP, Amaravati 522240, India; 3 Saha Institute of Nuclear Physics, Kolkata 700064, India; bikask.chakrabarti@gmail.com; 4 Economic Research Unit, Indian Statistical Institute, Kolkata 700108, India; 5 Department of Physics, Raghunathpur College, Raghunathpur, Purulia 723133, India; asimghosh066@gmail.com; 6 Deptartment of Physics, Babeş-Bolyai University, 400347 Cluj-Napoca, Romania; mate.jozsa@ubbcluj.ro (M.J.); zoltan.neda@ubbcluj.ro (Z.N.)

**Keywords:** citation data, inequality indices, Lorenz curve, Gini index, Kolkata index

## Abstract

We study the inequality of citations received for different publications of various researchers and Nobel laureates in Physics, Chemistry, Medicine and Economics using Google Scholar data from 2012 to 2024. Citation distributions are found to be highly unequal, with even greater disparity among Nobel laureates. Measures of inequality, such as the Gini and Kolkata indices, emerge as useful indicators for distinguishing Nobel laureates from others. Such high inequality corresponds to growing critical fluctuations, suggesting that excellence aligns with an imminent (self-organized dynamical) critical point. Additionally, Nobel laureates exhibit systematically lower values of the Tsallis–Pareto parameter *b* and Shannon entropy, indicating more structured citation distributions. We also analyze the inequality in Olympic medal tallies across countries and find similar levels of disparity. Our results suggest that inequality measures can serve as proxies for competitiveness and excellence.

## 1. Introduction

The inequality of acquired resources among competing entities is a well-established empirical observation. It is particularly well studied in the context of income and wealth inequalities [1]. More than a century ago, Pareto formulated, based on empirical data, his 80-20 law, which states that 20% of people in a society posses 80% of the total wealth [2]. It has since been modified and used in a wide variety of contexts from managements (see, e.g., [3]) and infectious disease spreading [4,5] to physical systems undergoing (or are near) a phase transition (see, e.g., [6]). More recently, this principle has also been applied to scenarios involving asset accumulation among many competing entities, where such competitions are seemingly unrestricted. Unlike cases such as wealth distribution or disease spread, where inequality often signals underlying issues, in these scenarios, inequality emerges naturally and can even be desirable. Such examples include the inequality of the fractions of votes received by candidates in an election, the inequality of the incomes of producers from different movies [7], and also the inequality of scholarly citations of the publications of a researcher [8,9]. In particular, the distribution of citations among an individual’s publications has been found to follow a heavy-tailed form. Prior work suggests that the Tsallis–Pareto (TP, also known as Lomax II) distribution provides a good fit to the empirical citation data of many authors [8]. Earlier studies also noted the difference in citation patterns between successful researchers and others [10]. However, the reasons for citation inequality is sometimes also contributed by structural biases (see, e.g., [11,12]) and field-dependent citation patterns (see, e.g., [13]). At the level of journals, such inequality will affect the impact factors [14]. Given these contexts, there is almost no need for an external intervention in mitigating the emergent inequalities in these cases, hence the competitions can be allowed to evolve in an unrestricted manner.

On the other hand, it has also been suggested—within a limited scope—that scientific excellence, when defined through prizes and awards of high repute, are often accompanied by a high level of inequality in scholarly citations of different publications of the recipient of such prizes or awards [15,16]. It has been a long-standing quest to obtain a reliable metric to asses scientific excellence. In this work, we study the emergent inequality in scholarly citations and seek at least a robust correlation between scientific excellence (measured through wider recognitions) and an inequality of scholarly citations of the different publications of a researcher. Using Google Scholar citation data for 126,067 researchers (each having at least 100 publications), we show that measures of inequality of citations could be a useful indicator of excellence.

We also look at another system involving unrestricted competition, which is the distribution of medals in Olympic games. We show that a very similar nature of inequality exists even for this case.

## 2. Methods

**Figure 1:**
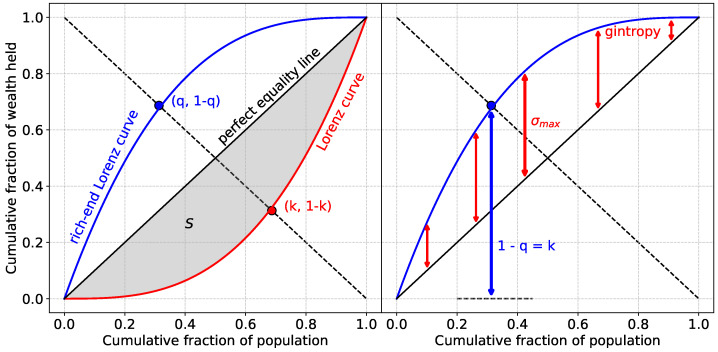
Different types of Lorenz curves and their relation to the studied measures: Gini index (*g*), Kolkata index (*k*), Pareto index (*q*), and maximum gintropy ($\sigma_{max}$). (**Left**) The Lorenz curve (in red) denotes the cumulative fraction of wealth or “assets” held by a cumulative fraction of the population when arranged in ascending order of their wealth. The rich-end Lorenz curve (shown in blue) is the same when the population is arranged in descending order of their wealth. If all agents have exactly the same wealth, then the Lorenz curve is a 45-degree straight line, called the perfect equality line. The area (*S*) between this line and the Lorenz curve (shaded region) is then one measure of inequality. Normalizing this area by the total area under the perfect equality line yields the Gini index (g=2S). The off-diagonal (dotted line) intersects the Lorenz curve at (k,1−k) and the rich-end Lorenz curve at (q,1−q), where 1−k fraction of the population holds *k* fraction of the total wealth, defining *k* as the Kolkata index. In terms of the Pareto index, a fraction *q* of the population possesses 1−q fraction of the wealth, implying q=1−k. (**Right**) Illustration of the “gintropy” measure, defined as the density function given by the difference between the rich-end Lorenz curve and the perfect equality line (red arrows). The maximum value of this function is denoted as $\sigma_{max}$, which, as shown in the figure, exhibits a strong correlation with 1−q=k, the Kolkata index.

As mentioned above, we quantify the inequality of “assets”, which we use as a generic term, in order to correlate with individual successes. Such quantification mechanisms already exist in the context of the quantification of wealth inequality. Particularly, if a total asset *A* is distributed among a group of *N* individuals, where the *i*-th individual has an $a_{i}$ amount of assets ($\sum_{i=1}^{N}a_{i}=A$), then one can first arrange the individuals in the ascending order of the assets possessed by them. Then, it is possible to define a Lorenz curve L(p) [17] that indicates the fraction of the total assets possessed by the poorest (in terms of the particular type of asset concerned) *p* fraction of the individuals. As is evident, the two limiting points of the Lorenz curve would be L(0)=0 and L(1)=1, with a monotonically increasing function in between. Clearly, if each individual had exactly equal assets (A/N), then the Lorenz curve would be a 45-degree straight line. This line represents perfect equality. On the other hand, any inequality in the asset distribution would necessarily make the curve concave, and the area opening up between the equality line and the actual Lorenz curve would then be a measure of inequality in the asset distribution (see Figure 1). Indeed, it is this area normalized by the area under the equality line that is defined as the Gini index [18], which is widely used and interpreted in Economics and various other fields [4,5,8] as a common measure of inequality. The mathematical definition could be written as $g=1-2\int_{0}^{1}L\left(p\right)dp$. A discrete version for the Gini index can also be similarly defined.

Another measure of inequality that we use here is the Kolkata index (*k*) [19] (see also, [20]), which is a generalization of the Pareto’s law. It is the fixed point of the complementary Lorenz curve $\tilde{L}\left(p\right)\left(\equiv 1-L\left(p\right)\right)$, $\tilde{L}(k)=k$. As such it says that the 1−k fraction of the richest individuals (persons/papers/countries,…) posses *k* fraction of the total assets (wealth/citation/medals, …; see Figure 1). Clearly, in the limit k=0.8, one obtains the 80−20 law. This index complements the Pareto index *q*, which describes how the wealth is distributed at different levels: for any fraction *q*, the richest *q* portion of the population owns 1−q of the total wealth. Extending this iteratively, the richest $q^{n}\times 100\%$ holds ${\left(1-q\right)}^{n}\times 100\%$ of the total wealth, with *n* being a non-negative real [21]. Unlike the Gini index, this has a more intuitive interpretation related to the reflection of the underlying inequality at least at its extreme end, i.e., the richest individuals (see, e.g., [22,23] for its application in kinetic wealth exchange model). The Kolkata index (*k*) exhibits a strong correlation with the maximum value of the “gintropy” measure ($\sigma_{\max}$) (see Figure 1). Gintropy itself is a density-like quantity, defined as the difference between the rich-end Lorenz curve and the perfect equality line (diagonal). Introduced in [24], gintropy establishes a connection between the Gini index and entropy, as implied by its name. Its entropy-like density characteristics were demonstrated, suggesting that the Kolkata index (*k*), given its high correlation with $\sigma_{\max}$, can be interpreted as an inequality measure with entropic properties.

To interpret the structure of asset distributions using information-theoretic measures, we considered Shannon entropy for continuous probability density functions (PDFs). For a PDF ρ(x), it is defined as S=−∫ρ(x)logρ(x)dx, capturing the uncertainty inherent in the distribution.

We analyzed individual citation distributions by fitting the Tsallis–Pareto (Lomax II) distribution, which has previously been shown to capture such data accurately [8]. The rescaled one-parameter probability density function is defined as

$$\rho \left(x\right)=\frac{b}{b-1}{\left(1+\frac{x}{b-1}\right)}^{-1-b},$$

where *x* is the citation count and *b* is the shape parameter. The corresponding cumulative distribution function,

$$F\left(x\right)=1-{\left(1+\frac{x}{b-1}\right)}^{-b},$$

was used to ensure a smooth empirical fit. For each researcher, the optimal *b* was obtained by scanning over a discrete set of values in the interval b∈(1,8), and minimizing the average of squared relative logarithmic differences between the empirical and theoretical cumulative distributions: $\frac{1}{N}\sum_{i=1}^{N}\left(\frac{\log F_{i}^{\exp}-\log F_{i}^{\mathrm{theo}}}{\log F_{i}^{\exp}}\right),$ where *N* is the number of data points. The fit was performed in logarithmic scale to accurately capture the tail behavior. This method was applied to all researchers in the dataset, including Nobel laureates, allowing for a comparison of the fitted *b*-parameters across groups.

Along the line of exploring the effect of the richest individuals in determining the inequality, we also looked at a quantity *Q*-factor [16], which is the ratio of the maximum value of asset possessed by the (richest) individual and the average asset value: $Q=max\left\{a_{i}\right\}/\left(A/N\right)$. In terms of citations, for example, this will be the ratio of the citation number of the highest cited paper of an individual and the average citation of all papers of that individual. Similar extensions can be made for Olympic medals as well.

Along with these, we also continued to use the more commonly used (for the case of citations), the Hirsch index (*h*) [25] that states *h* publications of the concerned individual have at least *h* citations each (see [26,27] for a review and other variants of the *h* index).

Now, the inequality indices are defined in a generic way such that it is possible to use these in a wider context. Particularly, we will focus on the inequality of citations of the publications of a given researcher and inequality of the medals received by different countries in the Olympic games (both summer and winter versions). As mentioned in the Introduction, both of these scenarios are examples of unrestricted competitions. We used Google Scholar data for 126,067 scientists (with more than 100 papers each) and 80 Nobel laureates in different fields during the period 2012–2024. The datasets are available in [28]. We also used the data for the Olympic medals received by different countries, both in the summer and winter versions of the games within the period 1896–2024 and 1924–2022, respectively; the data are available in [29,30].

## 3. Results

### 3.1. Inequality in Citations

We started with investigating the inequality in the citation data of the publications by individual researchers. In particular, we considered the citations received from the various papers of a researcher (including papers with no citations) who have at least 100 publications to ensure sufficient statistics, and measured the indices *g*,*k*,*h*,*Q*,*b* and *S* as described earlier. We carried out the same exercise for Nobel laureates in Physics, Chemistry, Medicine and Economics during the years 2012–2024, for the individuals with a public Google Scholar profile.

There are some prior observations that we need to first discuss in this context. It has been noted elsewhere that the citation inequality of different papers of a researcher, quantified through the Gini index, is surprisingly high [8]. However, if the successful researchers were considered (in terms of Nobel laureates, Fields Medalists, Boltzmann awardees, etc.), the inequality is, in general, even higher [7], indicated by both the Gini and Kolkata indices. Particularly, even though the ranges of these two indices are not the same [(0, 1) for *g* and (0.5, 1) for *k*], they tend to become equal (close to 0.87) for successful researchers. Firstly, this would mean that the citation inequality is more prominent among successful researchers. This observation could then be translated as indicators of excellence. Secondly, it is worth mentioning that the tendency of *g* and *k* to fluctuate around 0.87 has been found in studying the inequality of responses in many physical systems at or near a critical point [6]. It has been shown analytically and numerically that the crossing point of *g* and *k* signals an imminent system spanning response and that the value at the crossing point is rather weakly dependent on the underlying distribution function (assumed to be power law near the critical point). This in turn would then suggest that successful researchers could be near a self-organized critical state [6,31]. This is, of course, based on observational similarity with the behavior of known SOC systems.

**Figure 2:**
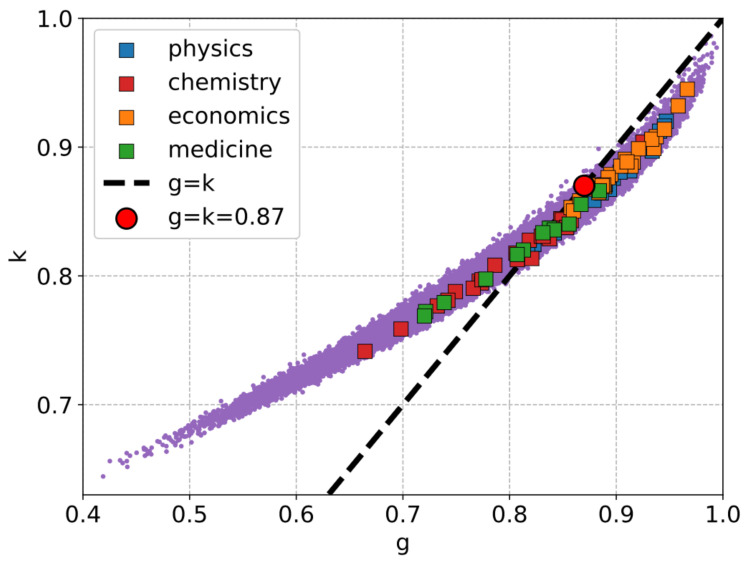
Illustration of citation inequality among an individual researcher’s papers, quantified using the Gini index (*g*) [18] and the Kolkata index (*k*) [19]. The background data (purple dots) represent 126,067 scientists with more than 100 published papers, based on Google Scholar records. Nobel laureates from 2012 to 2024 across different disciplines are highlighted using distinct colors. The dashed line corresponds to g=k, around which the data points for Nobel laureates tend to cluster. A seemingly universal critical point at g=k=0.87 is marked by a red dot. Note that k=0.8 for any author would suggest that 80% of citations come from 20% of the papers by that author (Pareto law).

**Figure 3:**
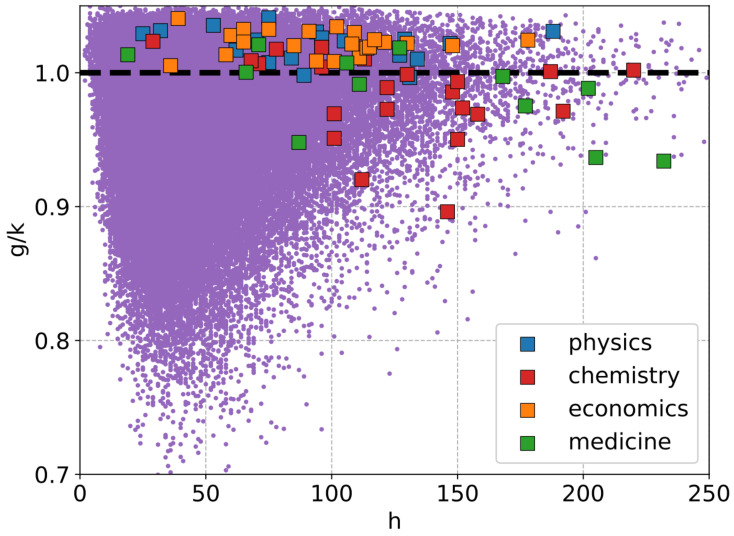
Illustration of the ratio between the Gini index (*g*) [18] and the Kolkata index (*k*) [19], representing citation inequality among individual researchers’ papers, plotted as a function of the Hirsch index (*h*) [25] for 126,067 researchers (purple dots). Nobel laureates from different disciplines are highlighted using distinct colors. All data are sourced from Google Scholar. The dashed line represents g/k=1, around which the Nobel laureate data tend to cluster (mostly within a range 1.00±0.03; see also Figure 6). Notably, data points from Physics and Economics tend to group together, distinct from those of Chemistry and Medicine. The former cluster generally exhibits g/k>1 with slightly lower *h* values, whereas the latter shows g/k<1 with comparatively higher *h* values.

**Figure 4:**
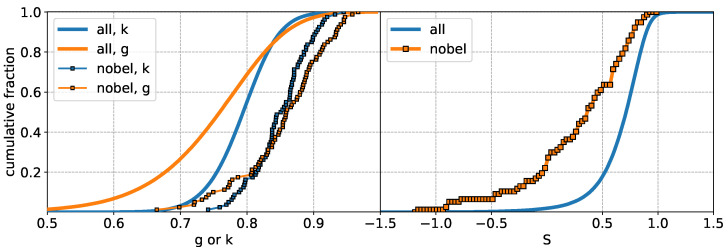
Cumulative fraction of scientists as a function of Gini (*g*, orange) and Kolkata (*k*, blue) indices (**left**), and Shannon differential entropy *S* (**right**). Nobel laureates are highlighted separately in both panels. The results indicate that Nobel laureates on average exhibit higher citation inequality and lower entropy compared to the general population.

In view of the above, we first plot *g* vs. *k* in Figure 2 for all 126,067 scientists and the 80 Nobel laureates in the abovementioned fields during the period 2012–2024. We also show the g=k line for reference. As can be seen, the data for the Nobel laureates are clustered around g≈k (indicating that their ratio would be close to unity, see Figure 3). This clustering, observed against the broader distribution of researchers, suggests that these inequality measures may serve as potential indicators for distinguishing highly successful researchers from the general population. The cumulative distributions of the inequality measures demonstrates this distinction (see Figure 4).

**Figure 5:**
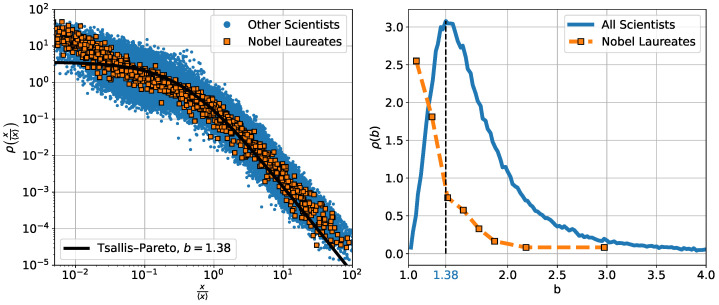
Analysis of Tsallis–Pareto fits to citation count data for Nobel laureates and the general scientific population. (**Left**) Citation distributions for all Nobel laureates (orange squares) and a random sample of 20,000 scientists from the general population (blue dots). The distributions overlap significantly and follow the same Tsallis–Pareto form, with the most probable fit corresponding to b=1.38 (see right panel). The Nobel laureates form a slightly narrower band of distributions. (**Right**) Distribution of fitted Tsallis–Pareto parameters *b* for the general population (blue) and Nobel laureates (orange). Nobel laureates tend to exhibit lower *b* values, clustering closer to b→1. The peak at b=1.38 is indicated in blue.

**Figure 6:**
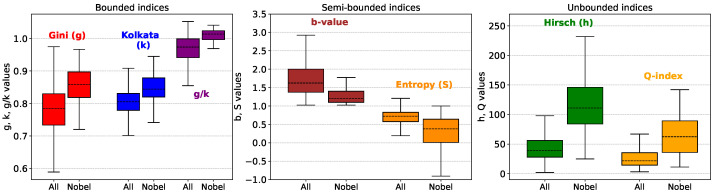
Box plot comparison of citation-based metrics for all researchers and Nobel laureates. The left panel shows bounded indices: Gini (*g*, red), Kolkata (*k*, blue), and their ratio g/k (purple), constrained to g∈(0,1), k∈(0.5,1), and g/k∈(0,2). The middle panel presents the Tsallis–Pareto shape parameter *b* (dark red), defined on (1,∞), and the Shannon differential entropy *S* (orange), defined on (−∞,∞). While formally unbounded, these indices span intermediate ranges in practice, narrower than *h* and *Q*. The right panel shows the unbounded indices: Hirsch index (*h*, green) and Q-factor (orange), both on (0,∞). Nobel laureates exhibit systematically lower values of *b* and *S*, and higher values in all other indices, compared to the general population. The separation is evident across all panels, supporting the relevance of these metrics in distinguishing scientific excellence.

We examined the citation distributions of Nobel laureates using the Tsallis–Pareto form introduced earlier. Their empirical distributions are well described by this model, similarly to the general population (see left panel of Figure 5). However, a notable distinction lies in the fitted values of the shape parameter *b*, which, for Nobel laureates, are systematically lower and tend to cluster near the lower bound b→1 (see also the middle panel of Figure 6). As shown in the right panel of Figure 5, the distribution of *b* values for Nobel laureates is clearly shifted compared to that of other researchers. This suggests that the parameter *b* may also serve as a useful indicator of scientific excellence, with Nobel laureates appearing close to the lower limit of the typical citation distribution.

It is worth discussing at this point the effectiveness of the well-known Hirsch index in making such distinctions. Particularly, in Figure 3, we plot *h* on the x-axis and g/k values on the y-axis. The Nobel laureates, as before, are indicated separately. The first pint to note is that the Nobel laureates have *h*-index values that are widely spread, but in terms of g/k, the ranges are narrow (see also left panel of Figure 6) and close to unity. There is, of course, the point that the *h*-index does not have an upper bound as such; indeed, it is often claimed to be related directly to the total number of citations ($N_{c}$) as $h∼\sqrt{N_{c}}$ [32]. It is a monotonically increasing quantity with time (unlike *g* and *k*) and often tends towards a high value for Nobel laureates (possibly due to a significant increase in $N_{c}$ after winning the prize). The second point here is that there is a clear abundance in the number of researchers near g≈k for whom the *h* index is higher. This might qualitatively indicate that, after all, such a correlation would imply placing high values of *h* in a similar footing as g≈k. Thirdly, an interesting observation is the clustering of Physics and Economics researchers together, distinct from the Chemistry and Medicine groups. The former predominantly exhibit g/k>1 with lower *h*-index values, whereas the latter tend to have g/k<1 and higher *h*-index values.

We have also calculated the *Q*-factor from the citation data. While the most probable value of *Q* for all scientists is around 20, for the Nobel laureates, it is significantly higher. Of course, like *h*, *Q* also does not have an upper bound.

In addition to the above indices, we computed the Shannon differential entropy based on the normalized citation distributions of individual researchers. The entropy values for Nobel laureates tend to be systematically lower than those of the general population, as shown in the right panel of Figure 4 (see also middle panel of Figure 6). This indicates that Nobel laureates tend to have citation distributions with lower disorder, as quantified by entropy, which may reflect a more concentrated impact across their publications.

It is worth noting the initially counterintuitive observation that the entropy associated with Nobel laureates tends to assume lower values (often negative) compared to that of the general population, while their inequality measures (e.g., the Gini index) are typically higher. One might expect that higher inequality would correlate with higher entropy. However, if we assume that the underlying distribution follows a Tsallis–Pareto form, this apparent paradox is resolved. For this distribution, both the Gini index and the differential Shannon entropy can be calculated analytically. Specifically, the Gini index is given by $G=\frac{b}{2b-1}$, and the differential Shannon entropy by $S=\frac{1+b}{b}-\log\left(\frac{b}{b-1}\right)$. The former is a monotonically decreasing function of *b*, while the latter increases monotonically, thereby explaining the observed behavior. Notably, the entropy becomes negative for b≲1.19, which also aligns with the observed behavior.

We have tabulated the inequality indices for some of the Nobel laureates (2020–2024) in Table A1 of Appendix A, where the narrow range of values for g,k for the individual laureates are apparent.

In view of the relative abundance of the population near g≈k for all scientists, we first establish that, on average, the inequalities are even higher among the Nobel laureates. The left panel of Figure 4 shows the plot of the cumulative fraction of all scientists as functions of *g* and *k* and it is then compared with the same plot for the Nobel laureates. It is clear that on average the inequality of the citations is higher among the Nobel laureates. This point in itself is not a conclusive statement on the effectiveness of *g* and *k* as indicators on excellence, since the average values of g,k,Q,h,b and *S* are all significantly different for the Nobel laureates, when compared with the overall data (see Figure 6).

**Figure 7:**
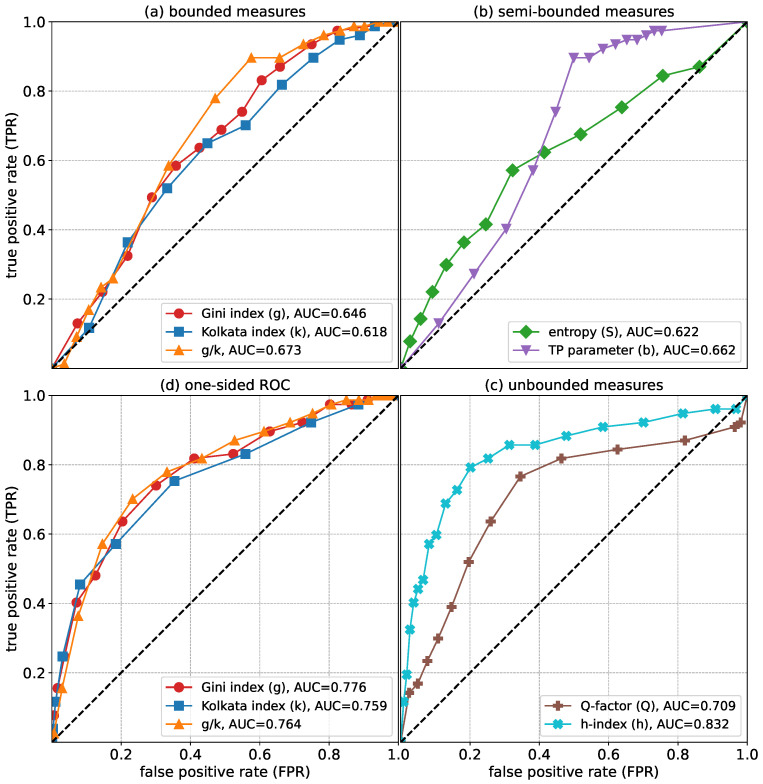
The ROC curves [33,34] are shown as follows: (**a**) for the bounded measures, viz., Gini *g*, Kolkata *k*, and also, for the ratio g/k; (**b**) for the semi-bounded measures, viz., the entropy *S* and TP parameter *b*; (**c**) for the unbounded measures, viz., the Hirsch index *h* and the *Q* factor; and (**d**) the one-sided ROC curves for the bounded measures. The straight dotted line (45 degree) indicates the complete random process of segregation. The area under the curve (AUC) of the ROC curves and this straight line gives a measure of the overall efficiency of any quantity in distinguishing Nobel laureates from the others.

A more standardized measure for the segregation of the Nobel laureates would be to construct a Receiver Operation Characteristic (ROC) curve [33] for each of these indices (or combinations thereof), such that an objective quantification of their relative success in distinguishing the Nobel laureates could be compared. In general, an ROC curve is a plot of True Positive Rate (TPR) with the False Positive Rate (FPR) in an attempted segregation as the parameter in question is gradually varied (see [34] for a review). In the present context, suppose we want to quantify the usefulness of any quantity *J* in performing the classification. We then measure the average of that quantity for all Nobel laureates. Then, we consider the standard deviation of *J* among the Nobel laureates and calculate the ratio of the fraction of Nobel laureates within the range 〈J〉−ΔJ to 〈J〉+ΔJ. Subsequently, we calculate the ratio of other scientists within the exact same range, where ΔJ=nσ, with σ being the standard deviation of *J*, and *n* is a number that we gradually increase. The fraction of Nobel laureates is then the TPR and the fraction of the others is the FPR, and we plot the points calculated for different values of *n* (see Figure 7), giving us the ROC curve. We also calculate the one-sided ROC curves, for which we set a threshold of *J*th value for an index, whereby the fraction of Nobel laureates above the threshold gives the TPR and the fraction of the other scientists falling above the threshold gives the FPR.

The ROC curve, by definition, is bounded between (0, 0) and (1, 1). Clearly, a 45-degree line is when the classifier works no better than random choices. The area under the curve (AUC) between the ROC and 45-degree line is then a measure of the performance of the classifier. This is then a standardized measure for any quantity, particularly the inequality indices, even though the quantities individually may have different ranges of their own. In this way, we can have a quantification of the comparison of performances of different scientometric metrics in segregating the Nobel laureates.

In what follows, we carry out systematic analysis in terms of the abovementioned ROC curves for all the parameters (inequality indices and the entropy-related parameters): *g*, *k*, *h*, *Q*, *b* and *S*. We also look at the one-sided ROC curve, but in this case, we only keep the bounded inequality indices. For unbounded parameters, setting the threshold is dependent upon the present range of the parameter, which in turn may depend upon the time elapsed since receiving the award, particularly for Nobel laureates.

As shown in Figure 7, we compare all quantities: *g*, *k*, their ratio g/k, entropy *S*, TP parameter *b* and the one-sided ROC for the bounded measures. Each of these measures serves as a relatively good indicator of citation inequality. However, the selection of a specific metric depends on the acceptable trade-off between false and true positives. Notably, g/k appears to perform slightly better than *g* and *k* individually, suggesting that the combined measure may capture inequality patterns more effectively. Also, the one-sided ROC plots perform very well compared to the other ROC plots.

### 3.2. Inequality in Olympic Medals

Finally, we turn to the question of the inequality of Olympic medals received by various countries in the summer and winter versions of the games over the period of 1896–2024 and 1922–2022, respectively. As mentioned before, in this case as well, a high level of competition is present. In the similar way as described before, one can construct the Lorenz curve and then calculate the inequality indices *g* and *k* for the medals received by different countries in a given year.

**Figure 8:**
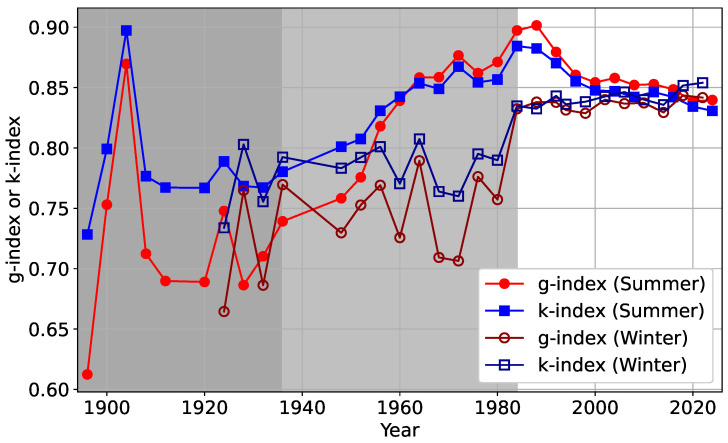
Event-wise (four-yearly) variations of the Gini (*g*) and Kolkata (*k*) indices for the number of Olympic medals won by the participating countries are presented for both Summer (filled markers) and Winter (empty markers) Games from 1896 to 2024 (see Table A2 and Table A3). Shaded regions indicate years with fewer than 50 participating countries, with lighter gray representing Winter Olympics and darker gray representing Summer Olympics.

In Figure 8, the time variation of *g* and *k* are shown for the Summer Olympic medals. Note that the number of participating countries have changed drastically over the years (see Table A2 in Appendix A). In more recent times, the inequality indices *g* and *k* have remained close to each other and around 0.85. A similar trend can also be observed for the Winter Olympic Games (see Table A3 in Appendix A).

These results suggest that irrespective of the underlying mechanism, an emergent inequality of near-universal nature arises for asset accumulation, when there is an unrestricted competition.

## 4. Discussions and Conclusions

Reduction in inequality is one of the Sustainable Development Goals that has been established by the United Nations since 2015 [35]. However, a little more than Pareto’s 80–20 (or self-organized critical) level of social inequality seems inherently tied to social competence and efficiency. An interesting question would be how far such high-level inequalities can be sustained in different spheres of social dynamics? Such questions are addressable, particularly in the contexts where accumulated asset inequalities are not tied to any dire consequences, or even sometimes a desirable phenomenon. If a steady state can then emerge out of the collective behavior of the competing individuals, it is then useful to inquire what the characteristics of such agents who accumulate a higher amount of assets are.

In this work, we tried to proceed along that direction, particularly in the context of scholarly citations among the papers of individual researchers and the Olympic medals (summer and winter) received by different countries in a year. The two situations are slightly different from one another. In the first case, i.e., the inequality of scholarly citations of different papers of many researchers, we note that researchers who are widely recognized as successful (Nobel laureates) have a higher inequality in the citations of their individual papers. More particularly, the Nobel laureates are disproportionately concentrated near the higher values of the inequality indices (Gini *g* and Kolkata *k* indices) when labeled with the inequality indices associated with them. We have made a quantitative analysis regarding the effectiveness of using the g,k and g/k indices in distinguishing Nobel laureates from the other scientists (see Figure 7). We have also studied the effectiveness, using the ROC and variants of it, for several other measures, such as the entropy *S*, TP parameter *b*, *Q* factor and Hirsch index *h*. It then suggests that inequality in this context could be a useful indicator of excellence. This is in line with the observation that scientific impact likely requires more than one index for quantification [36,37].

As mentioned before, the parameters we study can be divided broadly into three groups depending on their domain of values, bounded (*g*, *k*), semi-bounded (*S*, *b*) and unbounded (Q,h). Post-award, the unbounded indices can be disproportionately affected by the scientist’s recognition, whereas bounded and semi-bounded measures may remain more stable. Further quantitative analysis is required to validate this observation by examining the time evolution of these indicators and comparing pre- and post-award behavior. We also fitted the citation count distributions of individual researchers using the mean-normalized Tsallis–Pareto (Lomax II) distribution. A clear distinction was observed in the fitted shape parameter *b*, with Nobel laureates consistently exhibiting values closer to the lower limit b→1. This suggests that they lie near the boundary of typical citation patterns, in line with their position at the frontier of scientific excellence. Additionally, the Shannon differential entropy computed from normalized citation distributions was found to be systematically lower for Nobel laureates, indicating a more ordered structure in their citation profiles. It would also be valuable to explore the entropic properties of the Kolkata index, particularly through the maximum of gintropy, and examine its connection to the differences between Nobel laureates and other scientists.

We then also look at the case of inequality in winning the Olympic medals (both in the Summer and Winter Olympics) among the participating countries. Note that the Olympic Games is already the most competitive athletic event. Therefore, arguing along the same lines as above, one would expect the medal distribution to be highly unequal, which is exactly what we find, especially when the number of participating countries is large. The data on the Olympic medal inequalities should then be compared with the data points for the Nobel laureates in the citation case. In contrast, the data for the other scientists may resemble the medal tally of less-competitive sporting events, where inequality is not as pronounced as in the Olympics. It is difficult to conceive of such an example in a similar scale of participation, and we did not investigate along this line.

Finally, a couple of more points need to be noted for the citation analysis. Firstly, the number of Nobel laureates is restricted by other conditions than just excellence. There are only a fixed maximum number of laureates in a field in a year, which is not awarded posthumously, among other factors. This means that there could be many scientists in the range of citation inequality as that of the Nobel laureates who are just as excellent. Therefore, the false positive we mentioned earlier is not necessarily “false” in terms of distinguishing excellent researchers. A more detailed analysis of the scientists who fall in this range could be a fruitful future direction of research. Finally, an intuitive understanding as to why such citation inequalities are seen for Nobel laureates could be argued in the following way: If many papers of a researcher receive a similar level of (high) citations, a more probable explanation for that could simply be the higher rate of publications in their particular field rather than all those papers being outstanding. This situation would result in a high *h*-index but low g,k and *Q*. We do not, however, see this for Nobel laureates. In this case, the works awarded Nobel prizes would have received much more attention (even prior to the award) than other works of the laureates. There could of course be individual exceptions. The question of not observing extreme inequality (g,k near unity) for Nobel laureates is more subtle. It could be expected that except for the Nobel-winning work, an outstanding researcher such as a Nobel laureate would have made some other important contributions as well that would receive good citations, thereby preventing the inequality to reach an extreme level.

In conclusion, we find that emergent inequality is a good indicator of high competitiveness and excellence. We demonstrate this through extensive data analysis for scholarly citations and Olympic medal tallies over the years.

## Data Availability

The data used in this manuscript are available freely in https://figshare.com/articles/dataset/Data_for_article_i_Does_Excellence_Correspond_to_Universal_Inequality_Level_i_/28827314 (accessed on 19 April 2025).

## Abbreviations

The following abbreviations are used in this manuscript:

SOC	Self-Organized Criticality
ROC	Receiver Operation Characteristic
AUC	Area Under the Curve
TPR	True Positive Rate
FPR	False Positive Rate
TP	Tsallis-Pareto

## Appendix A

In this Appendix, we list the values of inequality indices of the citations of some of the Nobel laureates (between 2020 and 2024) and the inequality of medal tallies in the Summer and Winter Olympic Games during the periods 1896–2024 and 1924–2022, respectively.

**Table A1:** The table shows various statistical values for Nobel laureates who have Google Scholar profiles and won a Nobel Prize between 2020 and 2024.

Name	Year	Sub.	NP	NC	h	Q	g	k
Alain Aspect	2022	phys	757	40,141	75	122.893	0.9337	0.8969
Anne L Huillier	2023	phys	504	34,777	84	77.1796	0.8469	0.8377
Anton Zeilinger	2022	phys	1098	113,609	147	71.3036	0.8897	0.8707
Ardem Patapoutian	2021	med	184	50,057	87	11.2758	0.7387	0.7793
Benjamin List	2021	chem	337	48,209	101	28.4021	0.7492	0.7878
Carolyn R. Bertozzi	2022	chem	1000	97,852	150	37.1544	0.8072	0.8126
Daron Acemoglu	2024	eco	1268	256,141	178	94.1268	0.9101	0.8885
David Baker	2024	chem	2503	184,477	220	62.2481	0.8388	0.8372
David Card	2021	eco	663	99,436	114	43.0442	0.8924	0.8763
David W.C. MacMillan	2021	chem	560	81,172	130	62.8993	0.8297	0.8308
Demis Hassabis	2024	chem	162	194,682	96	28.6888	0.8480	0.8444
Emmanuelle Charpentier	2020	chem	269	59,389	62	94.3811	0.9315	0.9012
Ferenc Krausz	2023	phys	1117	87,620	129	86.0893	0.8860	0.8644
Gary Ruvkun	2024	med	343	71,629	111	34.5301	0.8130	0.8201
Geoffrey Hinton	2024	phys	724	905,074	188	138.225	0.9406	0.9124
Giorgio Parisi	2021	phys	1115	108,250	134	117.507	0.8419	0.8334
Guido W. Imbens	2021	eco	348	110,070	101	26.0981	0.8747	0.8674
James Robinson	2024	eco	808	123,197	102	123.98	0.9452	0.9138
Jennifer A. Doudna	2020	chem	841	143,756	159	121.594	0.8586	0.8431
John F. Clauser	2022	phys	133	21,317	32	64.0487	0.9452	0.9164
John Hopfield	2024	phys	303	92,855	94	93.1922	0.8936	0.8672
John Jumper	2024	chem	72	58,763	29	40.8523	0.9250	0.9038
Joshua D. Angrist	2021	eco	400	101,784	91	107.176	0.9159	0.8884
Katalin Kariko	2023	med	222	29,678	66	20.0473	0.8373	0.8350
Michael Houghton	2020	med	533	60,722	106	89.2609	0.8418	0.8357
Morten Meldal	2022	chem	409	31,525	68	136.415	0.8208	0.8135
Moungi G. Bawendi	2023	chem	971	173,369	187	71.702	0.8315	0.8308
Paul R. Milgrom	2020	eco	383	116,246	85	41.2521	0.9085	0.8905
Robert B. Wilson	2020	eco	285	35,042	58	41.9753	0.8824	0.8707
Simon Johnson	2024	eco	849	90,468	65	178.742	0.9666	0.9449
Svante Paabo	2022	med	581	144,899	177	66.567	0.7777	0.7975
Syukuro Manabe	2021	phys	290	48,232	89	36.7611	0.8228	0.8244
Victor Ambros	2024	med	180	71,607	71	47.1312	0.8842	0.8661

**Table A2:** The table shows various statistical values for Olympic medals (Summer Olympics) won by different countries from 1896 to 2024.

Year	Participating Countries	Total Medals	h	Q	g	k
1896	14	122	6	5.78	0.6124	0.7283
1900	26	284	6	10.65	0.7530	0.7992
1904	12	280	4	11.51	0.8696	0.8973
1908	22	324	8	10.36	0.7124	0.7766
1912	28	317	8	5.95	0.6898	0.7672
1920	29	449	11	6.35	0.6890	0.7669
1924	44	392	10	11.36	0.7478	0.7888
1928	46	356	10	7.39	0.6864	0.7684
1932	37	370	10	11.30	0.7103	0.7672
1936	49	422	12	11.97	0.7392	0.7803
1948	59	443	12	11.38	0.7583	0.8010
1952	69	459	11	11.59	0.7757	0.8074
1956	72	451	12	15.86	0.8180	0.8308
1960	83	461	10	18.77	0.8391	0.8425
1964	93	504	12	17.90	0.8583	0.8536
1968	112	527	13	22.94	0.8586	0.8489
1972	121	600	13	20.13	0.8766	0.8673
1976	92	613	12	18.96	0.8620	0.8543
1980	80	631	12	25.03	0.8712	0.8567
1984	140	688	13	35.66	0.8973	0.8844
1988	159	739	14	28.58	0.9015	0.8824
1992	169	815	16	23.36	0.8795	0.8703
1996	197	842	16	23.75	0.8605	0.8552
2000	199	927	16	20.06	0.8543	0.8477
2004	201	926	16	22.03	0.8579	0.8470
2008	204	958	16	23.97	0.8521	0.8423
2012	204	960	16	22.21	0.8528	0.8463
2016	207	972	17	25.89	0.8485	0.8421
2020	206	1080	17	21.66	0.8385	0.8343
2024	207	1044	15	25.10	0.8397	0.8307

**Table A3:** The table shows various statistical values for Olympic medals (Winter Olympics) won by different countries during the period 1924–2022.

Year	Participating Countries	Total Medals	h	Q	g	k
1924	16	49	4	5.55	0.6645	0.7339
1928	25	41	4	9.15	0.7649	0.8029
1932	17	42	3	4.86	0.6863	0.7555
1936	28	51	4	8.24	0.7696	0.7924
1948	28	74	6	5.30	0.7297	0.7832
1952	30	67	5	7.16	0.7527	0.7922
1956	32	72	6	7.11	0.7691	0.8010
1960	30	83	6	7.59	0.7257	0.7704
1964	36	103	7	8.74	0.7894	0.8074
1968	37	106	7	4.89	0.7093	0.7639
1972	35	105	6	5.33	0.7064	0.7600
1976	37	111	6	9.00	0.7762	0.7951
1980	37	115	6	7.40	0.7572	0.7898
1984	49	117	6	10.47	0.8324	0.8347
1988	57	138	7	11.98	0.8380	0.8325
1992	64	171	7	9.73	0.8378	0.8431
1994	67	183	8	9.52	0.8314	0.8362
1998	72	205	10	10.19	0.8287	0.8383
2002	77	237	9	11.70	0.8400	0.8426
2006	80	252	11	9.21	0.8367	0.8463
2010	82	258	10	11.76	0.8373	0.8400
2014	88	284	10	8.68	0.8294	0.8357
2018	92	307	12	11.69	0.8435	0.8517
2022	91	327	13	10.30	0.8417	0.8540
